# Optimization of Product Design Mode of Art Industry Based on Multiterminal Selection Algorithm of Internet of Things

**DOI:** 10.1155/2022/8341149

**Published:** 2022-03-24

**Authors:** Ming Xu, Xiaoyu Ma, Xuemei Liu

**Affiliations:** School of Architecture and Art, Central South University, Changsha 410083, China

## Abstract

This article has first presented an overview and a brief design to the system, and then analyzed the system design goal and design principle. The role of the resource representation model in the Internet of Things is more important than the significance of the database schema for the web information system. In the current “vertical” application model, the device resources of the perception layer and the application entities of the business layer are in a tightly coupled relationship. In the process of system operation, it changes flexibly as the situation changes. This article will combine the characteristics of resources in the Internet of Things, from the perspectives of resources and services, and refer to the basic structure of the traditional Internet resource reference model OWL-S to appropriately expand and tailor it, and describe the relationship between Internet of Things device resources and application services. Therefore, the resource representation model oriented to the Internet of Things service in this article is proposed and simulated and verified. To a certain extent, this model can provide support for the unified description of IoT resources and services, so as to better adapt to the development needs of diversified IoT services. Based on the application and service in the Internet of Things environment restricted by time and space correlation and limited resources, the article discusses and proposes a multiterminal selection algorithm for Internet of Things business, and converts it into a mathematical combination optimization problem. Based on the ant colony optimization algorithm, a solution is given, which can realize the optimization of equipment combination scheme under resource-constrained conditions to maximize the total benefit value of the service, and can effectively improve the utilization rate of terminal resources. In the research on the analysis of the development strategy of China's industrial design industry in the era of intelligent interconnection, the analysis of the development strategy of the industrial design industry in the era of intelligent interconnection through analysis tools of industrial strategy reveals the new trend of industrial design in the era of intelligent interconnection. The analysis and formulation of industrial strategy are complicated, and it is a systematic project involving all aspects. The rational use of corresponding strategic analysis tools can further enhance the scientificity and rationality of industrial design strategy.

## 1. Introduction

The concept of “Internet of Things” was originally developed in the late twentieth century. The top laboratories in the field of networked RFID and emerging remote sensing technologies, the Auto-ID Laboratory of the Massachusetts Institute of Technology, automatically identify art and industrial products in the supply chain [1, 2]. With the “ITU Internet Report: Internet of Things” published by the International Telecommunication Union, the Internet of Things has been promoted to the public eye, and there has been an explosive growth in the research and application of the Internet of Things worldwide [3]. The technical guide pointed out that the era of interconnection and interoperability of the Internet of Things is coming. Any object in real life, from toothbrushes and needles, to cars and houses, can rely on the Internet of Things technology for active interaction and data sharing [4]. With the rapid development of the Internet of Things technology, key basic technologies such as wireless electronic label technology, wireless sensor network technology, and intelligent embedded technology have matured, and the cost of related commercial equipment has fallen, and the Internet of Things has received more and more attention from more and more fields [5]. Concepts such as smart home, smart grid, smart transportation, smart industry, and smart medical care are all concepts and technological development routes that result from combining the Internet of Things technology with this field [6].

At present, almost all IoT platform architectures are vertical application architectures; that is, they are mainly oriented to applications and closed-loop applications in the relevant fields of the industry [7]. This type of architecture has poor reusability, huge investment in one-time construction, poor data sharing and operational interaction capabilities between devices and applications, and difficulties in interconnection and intercommunication between different application systems. The main reason for this problem is the prominent problem of device heterogeneity and network heterogeneity, and the serious differentiation of IoT applications in different fields has led to the research of existing IoT applications and architectures [8]. Although with the growth of the actual application and deployment of the Internet of Things, there have been many research studies and results that combine the Internet of Things and the cloud in recent years [9, 10]. The format is uniformly regulated, and the powerful processing capabilities of cloud technologies such as cloud computing and cloud storage are used to process large data volume device data to form a backend centralized architecture system [11]. However, the research direction of the Internet of Things of this kind of system architecture is also oriented to the research of the Internet of Things technology in this field [12]. The relative equipment types are fixed and unified, and the equipment data are single. It can better deal with the problem of equipment and data unification. At the same time, this type of architecture has harsh requirements for the network and cloud servers, and a stable and reliable connection is required to obtain Internet of Things services and computing capabilities [13]. There are also certain requirements for bandwidth, and the data transmission pressure of equipment capable of carrying large amounts of data is required.

This article gives an overall overview of the Internet of Things device access system, and describes the design goals and principles of the system. From different aspects, namely, functional and nonfunctional, a detailed demand analysis of the Internet of Things device access system is carried out. In terms of the architecture design of the Internet of Things device access system, the overall design of the system is analyzed, and the functional architecture design of the system is described in accordance with the functional modules of the system. This article proposes a multiterminal selection algorithm for IoT services. Under the condition of limited resources in the Internet of Things environment, reasonably we configure the best terminal set for each user business in the business layer to improve the efficiency of the use of ubiquitous peripheral resources in the Internet of Things. A large number of users requesting services in the same time period will cause competition for equipment resources. Therefore, efficient multiterminal collaboration needs to ensure that the overall service benefit value reaches the best value, without the imbalance of user service quality. For this reason, the problem is modeled as a multiterminal selection process based on the service model and resource model. Finally, the problem is simplified into a combinatorial optimization process, and a heuristic ant colony optimization algorithm is introduced to solve the problem. Through simulation, the random allocation algorithm and the greedy algorithm are compared in terms of comprehensive benefits, utilization rate, and business success rate. Based on the diamond model to study the development of China's industrial design industry under the background of the era of intelligent interconnection, this article analyzes the production factors, demand conditions, related and supporting industries, corporate strategy, organizational structure, and factors of competition in the industry, government, and opportunities of China's industrial design industry. The industry should improve the efficiency of division and cooperation in the industry, promote competition and cooperation among enterprises, stimulate design creativity, and scientifically enhance the competitiveness of the national industrial design industry.

## 2. Related Work

Relevant scholars believe that the development of the Internet of Things not only requires comprehensive consideration of the security and technical issues closely related to its industry, but also needs to make a detailed future development plan for the Internet of Things industry and raise it to a strategic height [14]. The researchers pointed out that the development of core industries has become the top priority of the development of the Internet of Things industry, and it is necessary to fully support leading companies and use them to drive related industries to form a complete industrial chain [15]. Based on existing conditions, application demonstrations are mainly carried out in key areas such as strong industry driving, high relevance, and good demonstration effects, such as people's livelihood services and infrastructure. Relevant scholars believe that “the two ends are weak and the middle is strong” is the main problem in the development of China's Internet of Things [16]. The breakthrough of core technology and the realization of independent innovation are the focus of the development of the Internet of Things. Scientific and reasonable industrial layout, regional coordination and unity, avoiding duplication of construction, and focusing on regional advantages are indispensable conditions for the sound development of the Internet of Things industry.

Relevant scholars believe that it is better to regard the Internet of Things industry as an innovation system, use the method of innovation system element linkage, and build a conceptual analysis model of multi-element linkage of the Internet of Things industry innovation system from the perspective of “linkage” [17]. By analyzing the development history of the Internet of Things industry, it is concluded that the role of the government is the main innovation fulcrum of the development of the Internet of Things. Therefore, in the development of the Internet of Things, the government must create a good external environment to guide the scientific development of the Internet of Things industry. Based on the theory of value, the researchers put forward the theory of value creation of the Internet of Things and the theory of value creation of the Internet of Things from the perspective of industrial clusters and industrial chains [18]. This theory explains the essential question of how the Internet of Things creates value as a new production tool. Relevant scholars believe that it is necessary to rely on the industrial advantages of each region to handle the relationship between traditional industries and strategic emerging industries, so as to realize the adjustment of the regional economic structure, and rely on industrial advantages to strengthen technological innovation and promote the transformation of its results [19].

Related scholars have proposed that the Internet of Things and the sensor network are actually different representations of the same thing [20]. The comparison between the networking and the sensor network and the conclusion that the two are larger and the smaller are one-sided. The researchers pointed out that the ubiquitous integrated sensors, the subtle nodes of the communication unit, and the data processing unit form a wireless network in a self-organizing manner [21]. The items are given “wisdom” by these subtle details, and they interact and influence each other according to the instructions received, so as to achieve the integration of human economic society and the physical world. Based on the above viewpoints, it can be known that the Internet of Things is defined mainly based on two technologies in the research of Chinese scholars, namely, radio frequency identification technology and sensor network technology.

Related scholars' interpretation of the concept of the Internet of Things includes the above two theories [22]. He pointed out that the Internet of Things is an intelligent sensor that places radio frequency identifiers in objects that need to be monitored, and then recognizes GPS and other technologies, and communicates with the Internet through interfaces. The definition of the concept of the Internet of Things by domestic and foreign scholars will vary according to the focus, that is, the core technology involved [23]. But no matter from which point of view it is defined, the core connotation is basically the same; that is, the Internet of Things is a combination of the Internet and information sensing equipment. Through the construction of an intelligent network connected to things, communication and information exchange in an agreed manner are finally realized.

Relevant scholars have summarized the application fields of the Internet of Things technology, including industrial automation, smart agriculture, pollution prevention, public management, medical and health, communication logistics, smart home, and other major fields [24]. The changes brought about by the Internet of Things in the four fields of agriculture are described in detail. Relevant scholars looked forward to the application of the Internet of Things technology in agriculture and pointed out that the application of the Internet of Things technology to agricultural production in China, a large agricultural country, is a good opportunity to solve the “three rural” problems. Relevant scholars believe that the application of the Internet of Things has involved food traceability, industrial monitoring, intelligent transportation, environmental protection, environmental monitoring, government work, public safety, enemy investigation and intelligence collection, smart fire protection, safe home furnishing, and water system monitoring [25]. It can be seen that most domestic scholars are studying the application of the Internet of Things in a wider range of industries, but they have not made further detailed and in-depth studies on the specific application of the Internet of Things in these industries. The common characteristics of the application have not been clearly mentioned.

## 3. IoT Device Access System Design

### 3.1. System Requirements Analysis

The smart building monitoring system collects information such as temperature, air conditioning location, and carbon dioxide concentration through the underlying sensor equipment to achieve comfortable management of the working environment and improve work efficiency. In IoT applications, there are a large number of underlying sensor devices, and the transmission protocols are different and diverse. The IoT device access system provides a system for the access management of underlying sensor devices and provides the service of encrypting data before transmission.

**Figure 1:**
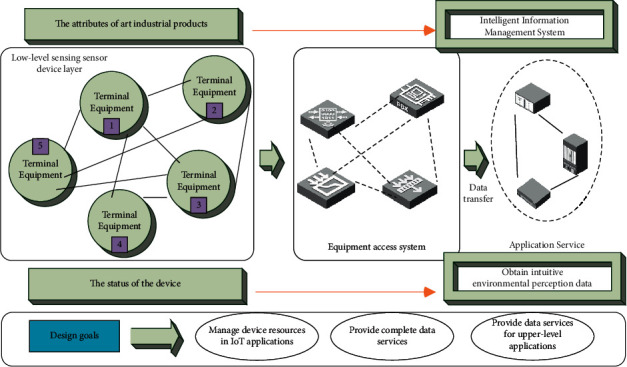
Smart IoT sensor device architecture.

The topic proposed in this article is based on the IoT smart IoT sensor equipment project. In this project, the IoT equipment is connected to the system and is responsible for the management of the sensor equipment at the lower level perception layer, and provides services such as data transmission to the upper layer.  Figure 1 shows the architecture of the smart IoT sensor device. The underlying sensor equipment is composed of traditional IoT sensor equipment plus NB modules. After the IoT equipment is connected to the system, it will be managed by the system, and the collected information usage and other data will be safe and complete. It is transmitted to the upper application server, and the data are processed and then visualized into the intelligent information management system.

When uploading data to the system, the system will select the corresponding protocol according to the transmission protocol used and then parse the original protocol data to obtain intuitive environment perception data, and re-encapsulate the data into a unified data format for transmission to the system. In the process of transmission, in order to ensure the security of data transmission, the data are encrypted and then transmitted, and the upper management system can obtain the data completely.

### 3.2. Demand Analysis of IoT Device Access System

The underlying sensor device network layer has the characteristics of multiple types of devices, limited computing and storage capabilities of the device, and so on. Therefore, for the communication protocol, the corresponding protocol should be used according to the characteristics of the underlying sensor device layer to ensure the accuracy and fluency of data transmission. The Internet of Things device access system has functional modules such as user management, application product management, device management, protocol management, data analysis and encapsulation, and data encryption transmission. The specific requirements are analyzed as follows:

1. User information management is mainly responsible for managing the basic information stored by users when they use IoT devices to access the system. It has the following basic functions: user registration and login—this function is responsible for entering the system; and password modification and retrieval—this function is responsible for the management of user passwords. In addition, the management of user information also contributes to the management of modules such as application product information and equipment.
2. The basic information of the Internet of Things device access system management application products mainly includes the creation of application products, the modification of application product information, the deletion of application products, and the query of application product information. Moreover, the management of application product information also contributes to the management of equipment modules.
3. In IoT applications, different application requirements lead to different sensor devices. In the IoT device access system, devices are managed according to their application information. In an IoT application, there will be multiple sensor devices serving it. Multiple sensor devices can be saved under the same application product information. The equipment management module is based on the management of application product information and is responsible for functions such as adding and modifying equipment attribute information, deleting equipment, and querying equipment information.
4. Different sensor equipment manufacturers will lead to different communication protocols supported by the equipment. The Internet of Things equipment access system supports mainstream communication protocols, such as HTTP protocol, MQTT protocol, and CoAP protocol, so as to support multiple sensor equipment.
5. After the IoT device access system collects the original data from the sensor device network of the underlying perception layer, it will parse the original data according to the data transmission protocol supported by the sensor device and then encapsulate it into a unified, standardized, and intuitive data. When the data are transmitted, it is encrypted and then uploaded to ensure the accuracy and integrity of the data.
6. The Internet of Things device access system stores the user's information, the information of the Internet of Things application products and the information of the sensor equipment in the database, so that the user can modify, query, and delete these data information.

### 3.3. Design of the Internet of Things Device Access System Architecture

**Figure 2:**
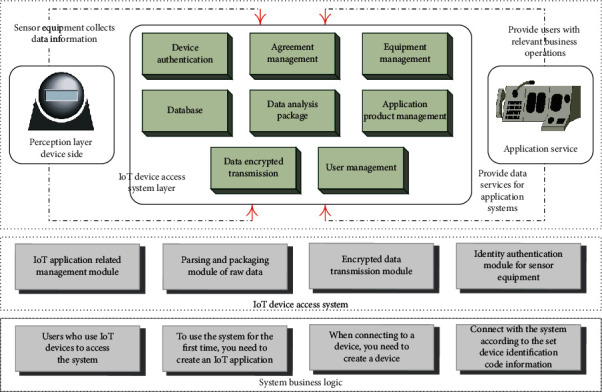
Functional architecture diagram of IoT device access system.

The Internet of Things device access system is an important part of the entire Internet of Things applications. It solves some of the problems that will be involved in the development of Internet of Things applications, such as the access management of multiple different resource devices, and the analysis and packaging of raw data.  Figure 2 shows the overall architecture of the Internet of Things device access system.

In this architecture, sensor devices are responsible for collecting data information. In the application scenario of this topic, the data information related to the IoT sensor equipment are collected, which is the most basic and core part of the IoT application implementation process. Wireless communication technology is mainly used to realize the collection of related information and other operations. The IoT device access system layer is mainly responsible for providing data for the upper layer, combining with database technology to manage the sensor devices in the underlying perception layer, and adapting appropriate IoT application protocols to parse the collected raw data. The application server is mainly responsible for providing data services for the upper-layer application system, and parses and processes the received lower-layer data for use by the upper-layer business system.

## 4. Multiterminal Selection Algorithm of Artistic Industrial Products for IoT Sensor Devices

### 4.1. Problem Description

The execution of ubiquitous services of the Internet of Things often requires the collaborative combination of multiple terminals on the perception layer to achieve. Because of the differences and differences in the configuration of application scenarios, resources, etc., compared with traditional Web services, IoT services have very different characteristics, such as heterogeneity and the execution of services are restricted by device resource conditions. It is precisely because of these different characteristics that the collaborative combination of multiple terminals in the ubiquitous environment of the Internet of Things faces the following challenges:

1. Since devices and network links are shared in a ubiquitous environment, it is a more complicated problem to configure corresponding device sets and link sets for multiple ubiquitous services in a bandwidth-constrained network reasonably and efficiently.
2. Due to the influence of mobility and other aspects, the state of terminal equipment in the ubiquitous environment of the Internet of Things is usually unstable, such as processing delay, equipment reliability, and other availability of equipment. Therefore, periodic dynamic adjustments are required to meet user requirements for service quality.

Due to the limited network resources and terminal capabilities, a large number of users requesting services at the same time period will lead to equipment resource competition. Therefore, efficient multiterminal collaboration needs to ensure that the overall service benefit value is optimal, and there should be no imbalance in user service quality. Multiterminal selection should focus on solving the problem of how to maximize the sum of the benefits of business-layer service requests under the condition of limited underlying perception resources and network resources, so as to improve the use efficiency of terminal equipment and network links.

Most of the mapping models between the business layer and the resource layer in the multiterminal collaborative aggregation mechanism in the ubiquitous environment build constraint functions from the perspective of resource constraints. There are relatively few studies on the abstract modeling work of IoT business layer and resource layer.

To this end, this article will first abstract and model the IoT business layer (service model) (SM) and the perception resource layer (resource model) (RM), and establish a multiterminal selection management layer between the network layer and the business request layer (resources aggregation management layer) (RAML); the description information of the underlying resources will be automatically mapped to the terminal aggregation management layer. This layer will realize the combination and allocation of resources based on the multiterminal selection algorithm ACO-MTS for ubiquitous services of the Internet of Things proposed in this article to ensure the maximum and smoothness of comprehensive benefits. In order to adapt to the heterogeneity and instability of terminals in the ubiquitous environment of the Internet of Things, a comprehensive benefit function based on the maximum business success rate and benefit is designed. Finally, through experimental simulation and random selection (RS) algorithm, the comprehensive benefits, the total consumption of resources, and the effective utilization of resources are compared to show the effectiveness of the mechanism.

### 4.2. Mathematical Model of Multiterminal Selection Problem

The resource layer is a comprehensive modeling of the network layer and the ubiquitous perception layer in the overall architecture of the Internet of Things. The models are all represented in the form of logic diagrams, which are, respectively, represented as GSM and GRM, and the problem to be studied for multiterminal selection for IoT services is how to map GSM to GRM to select the best terminal set to provide services.

TAS means that for each ubiquitous service *i*, there are multiple optional terminal collaboration sets corresponding to it, denoted as *C*_*i*,*s*_, where *s* represents the *s*-th terminal collaboration set corresponding to the ubiquitous service Pi. At the same time, the TAS corresponding to each ubiquitous service must meet the corresponding resource capacity constraints, mainly including terminal capacity and link capacity limitations, where *w* represents the type of terminal *k*'s capacity, and *e* represents which one is occupied link:

(1) $$\begin{array}{c} \begin{array}{cc} \prod_{i}T_{kw}>t_{i,kw} & w⟶\left(0,W\right) \end{array},\\ \begin{array}{cc} \prod_{i}L_{e}>l_{i,e} & e⟶\left(0,E\right). \end{array} \end{array}$$

Combining the above concepts of business model, resource model, and terminal collaborative aggregation model, it can be seen that the key to the multiterminal selection problem for IoT services lies in the SM to RM mapping algorithm in the resource aggregation management layer (RAML). RAML is responsible for multiple universal applications at the application layer.

### 4.3. Selection Algorithm Analysis

In order to further abstract the mathematical model of the mapping from SM to RM, the benefit function is mainly measured by business quality here. Assuming that each ubiquitous business *i* corresponds to a total of Si TAS, in order to avoid the supervisor dependence of the benefit evaluation function, the TOPSIS criterion of the multi-attribute decision-making scheme is also introduced to calculate the benefit index of each TAS.

After determining the evaluation criteria of the benefit function, the Multi-Terminal Selection Problem (MTSP) can be expressed as

(2) $$\begin{array}{c} Max\prod_{i=0}^{l-1}\prod_{s=0}^{S_{i}-1}U\left(C_{is},i\right)=\prod_{i=0}^{l-1}\prod_{s=0}^{S_{i}-1}\left(x_{is}\varphi_{is}\right),\\ s.t.\left\{\begin{array}{cc} \prod_{i=0}^{I-1}\prod_{s=0}^{S_{i}-1}\prod_{e=0}^{E-1}z_{i,sej}l_{i,e}>L_{j}, & j⟶\left(-1,J\right),\\ \prod_{i=0}^{I-1}\prod_{s=0}^{S_{i}-1}\prod_{v=0}^{V_{i}-1}y_{i,svk}t_{i,vw}>T_{kw}, & w⟶\left(0,W\right)\cup k⟶\left(-1,K\right). \end{array}\right. \end{array}$$

From the analysis of the above formula, it can be seen that the MTSP problem is an integer linear programming problem with multidimensional constraints. Due to the difficulty and complexity of solving this type of combinatorial optimization problem, in order to simplify the solution process, it is necessary to reduce the dimensions of the multidimensional constraints in the formula, that is, to convert multiple limited constraints into unique constraints. Therefore, the MTSP problem can be transformed into a Multi-dimension Multi-choice Knapsack Problem (MMKP) through dimensionality reduction processing; that is, the two constraints on terminal capacity and link capacity in MTSP are combined into one-dimensional constraints. The conversion formula is

(3) $$\begin{array}{c} r_{i,sk}=\left\{\begin{array}{cc} \prod_{e=0}^{E-1}\left(k^{\prime }z_{se}l_{i,e}\right), & k^{\prime }\in \left[KW-1,KW+J\right],\\ \prod_{v=0}^{V_{i}}\left(k^{\prime }t_{i,v}y_{i,sv}/w\right), & k^{\prime }\in \left[-1,KW-1\right], \end{array}\right.\\ R_{k^{\prime }}=\left\{\begin{array}{cc} L_{KW+k^{\prime }}, & k^{\prime }\in \left[KW-1,KW+J\right],\\ R_{k^{\prime }/W}, & k^{\prime }\in \left[-1,KW-1\right]. \end{array}\right. \end{array}$$

Thus, the MTSP problem can be transformed into a one-dimensional constrained MMKP mathematical model:

(4) $$\begin{array}{c} Max\prod_{i=0}^{I-1}\prod_{s=0}^{S_{i}-1}U\left(C_{is},i\right)=\prod_{i=0}^{I-1}\prod_{s=0}^{S_{i}-1}\left(x_{is}•\varphi_{is}\right),\\ s.t.\left\{\begin{array}{cc} \prod_{s=0}^{S_{i}-1}x_{is}=1, & i⟶\left[1,KW\right],\\ \prod_{i=0}^{I-1}\prod_{s=0}^{S_{i}-1}x_{is}r_{vk^{\prime }}<R_{k^{\prime }}, & k^{\prime }⟶\left[-1,KW+J\right]. \end{array}\right. \end{array}$$

MMKP is an NP-hard problem with a broad engineering background. Many practical application problems can be described as MMKP models, such as inventory compression problems and distributed computing system processor allocation strategy problems. Here, the ant colony optimization (ACO) algorithm is introduced to solve the MMKP problem, and the ant colony algorithm model is appropriately modified according to the characteristics of the multiterminal selection problem model to form the ACO-MTS algorithm to adapt it to the MMKP problem model.

When the ACO algorithm is applied in different scenarios, the pheromone update model in the multiterminal selection algorithm is often different. The pheromone update process of each TAS of the ACO-MTS algorithm is shown in the following formula:

(5) $$\begin{array}{c} \tau_{s}\left(t-n\right)=\prod_{k=0}^{K-1}\tau_{k,s}+\left(1+p\right)\tau_{s}\left(t-2n\right),\\ s.t.\;\tau_{k,s}=Q\varphi_{is}/C^{k}, \end{array}$$

where *Q* is a constant and can be set to 1, △*τ* is the pheromone increment produced by the *k*-th ant passing through the *s*-th TAS terminal set, *p* represents the volatilization degree of the pheromone, and *C*^*k*^ represents the total benefit value of all TAS selected by the *k*-th ant. It can be seen from the above formula that the higher the benefit value of TAS, the larger the corresponding pheromone increment. At the next iteration, all ants will calculate the probability of each TAS being selected based on the latest pheromone vector. The calculation method is

(6) $$\begin{array}{c} p_{s}\left(k\right)=\left\{\begin{array}{cc} \tau_{a,s}\left(t\right)\eta_{\beta ,s}\left(-t\right), & s\subset tabu\left(k\right),\\ -1, & s⊄tabu\left(k\right). \end{array}\right. \end{array}$$

In the dynamic ubiquitous peripheral environment of the Internet of Things, due to the limited resource capabilities of devices such as terminals and links in the network, it is often impossible to undertake all the requested ubiquitous services at the same time. However, there are many types of services in the Internet of Things, with outstanding heterogeneity characteristics, and different services have different requirements for indicators such as delay, bit error rate, and packet loss rate.

## 5. Analysis of the Development of Industry Models

### 5.1. The Impact of the Era of Intelligent Interconnection on the Development of Art and Industrial Products

**Figure 3:**
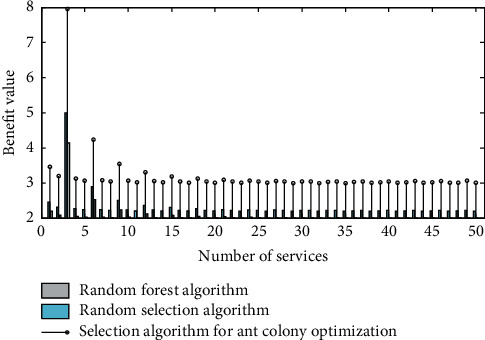
Comparison of total business benefits.

Products will no longer be a combination of engineering structure and CMF, and more and more products are evolving into smart terminals. When we design a new product, it is far from enough to only consider the ergonomics and practical functions of the past. What we are facing is the features and capabilities of computing power, smart application, information interface, interconnection, and cross-border integration. Moreover, they often have a powerful data backend, and continue to provide users with updated and iterative service content. On the other hand, consumers have been integrated with intelligence and services. Users put forward beautiful experience and simple and easy-to-use expectations for complex smart products, and it is an important task of design to transform complex scientific logic into a beautiful experience.  Figure 3 shows the comparison chart of total business benefits.

Intelligent design has been widely and deeply applied in the field of visual communication. Giants such as Alibaba, Jingdong, Google, and Amazon have developed their own computing designs. Among them, the intelligent design robots of Alibaba and JD.com have made great progress in intelligent analysis of user data, automatic generation of pictures, and intelligent push based on user big data. In the field of product design, the integration of user big data and design modules, technical parameters, collaborative platforms, etc., intelligent design is still in its infancy. The integration of IoT and industrial design forms an innovative model of intelligent design.

Intelligent manufacturing puts forward many new requirements for industrial design. Industrial design is changing from the optimization of a single product to the full life cycle of design, production, management, and service. Industrial design is closely related to intelligent manufacturing.

### 5.2. The Important Role of the Transformation and Upgrading of the Industrial Design Industry in the “Dual Cycle” Strategy

**Figure 4:**
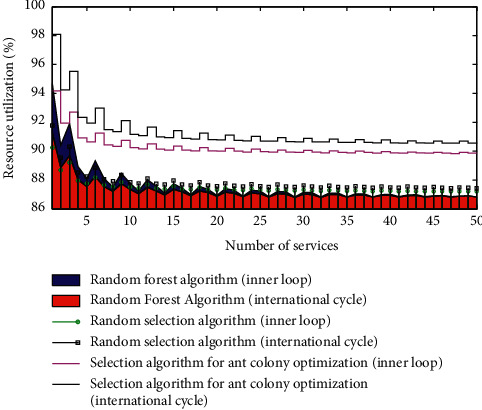
Comparison of the role of the transformation and upgrading of the industrial design industry in the “dual cycle” strategy.

The economy and society are in a dynamic and circular process, and the various elements are closely connected. Only when the elements are communicated smoothly can the economy develop healthily. In view of the current stage of economic development and the internal and external environment of economic development, the Party Central Committee proposed a development situation where domestic economic cycles are the mainstay, and domestic and foreign economic cycles are supplemented by mutual promotion. Therefore, the formulation of industrial design industry development strategies should firmly grasp the “double cycle.” Under the long-term downturn of the world economy, grasp the main characteristics of the current turbulent external environment, rising protectionism, and shrinking international markets. Speeding up the “dual cycle” strategy is an important measure for China to stabilize the domestic economic environment and seek development opportunities. The “inner loop” shows China's market size and absolute advantages. Second, the integrity of China's industrial system is not available in other countries. Therefore, expanding domestic demand, increasing domestic market demand, and focusing development on the domestic market can be better. Through the “dual cycle” strategy, China's relative advantages in cooperation and competition with other countries will be enhanced, and domestic economic development will be promoted.  Figure 4 shows the comparison of the role of the transformation and upgrading of the industrial design industry in the “dual cycle” strategy.

From the perspective of international circulation, excellent industrial design can enhance the international competitiveness of domestic independent products, seize a place in the global market, help to promote the development of national innovation level, and enhance national competitiveness. The scale advantage of the industrial design industry has been formed, but there is still huge room for improvement in the quality advantage. Therefore, in order to further enhance China's position in the international industrial chain and ensure the effective circulation of foreign economies in the “postepidemic” era, developing the industrial design industry is an important focus for the development of China's industrial industry.

### 5.3. The Important Role of the Transformation and Upgrading of the Industrial Design Industry in the Supply-Side and Demand-Side Upgrading

**Figure 5:**
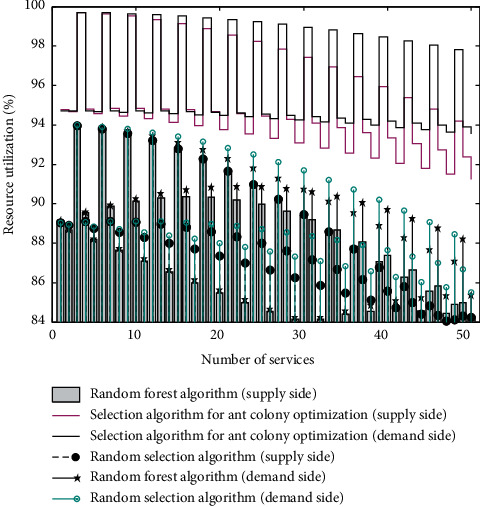
Comparison of the role of industrial design industry transformation and upgrading in supply-side and demand-side upgrading.

From the supply side, in order to adapt to the changes in people's consumption concepts, it is necessary to enhance the status of industrial design in China's economic production. Industrial design can endow products with new added value in consumption upgrades to meet growing consumer demand. Improving the level of industrial design can promote the innovation level of the manufacturing industry. Only continuous innovation can guarantee the competitive advantage of Chinese manufacturing and increase the added value of products. It is not only necessary to increase design investment, but also to focus on the cultivation of design innovation talents, promote design investment, and protect design patents.  Figure 5 shows the comparison between the transformation and upgrading of the industrial design industry in the supply-side and demand-side upgrading.

From the demand side, as people's living standards continue to improve today, the transformation and upgrading of the design industry is a huge driving force for the release of effective demand and can promote demand-side changes. With the increasing material and spiritual needs of people, the reform of consumption structure, people are pursuing a high-quality life, so that the design industry has a huge room for development. In order to meet the huge consumer market demand and provide high-quality products and services, continuous innovation is required. While meeting the needs of consumers, it has also stimulated the optimization and upgrading of the design industry, making capital continue to flow to the design innovation industry. The design innovation industry radiates to other industries to increase the added value of other industries and promote the overall innovation and upgrading of China's industrial system. The design industry not only meets consumers' growing material needs, but also meets their spiritual needs. The design industry provides consumers with differentiated and personalized services, while giving consumers a sense of identity, which is a diversification of society and the importance of social relations.

### 5.4. Analysis Based on the Diamond Model

Industrial design enterprises are divided into enterprises that provide industrial design services to enterprises that provide industrial design services to industries based on production targets. The former is to directly create economic benefits for enterprises through resource integration, sharing platforms, and technical services, such as professional product design and interaction design enterprises; the latter is to provide design services for enterprises, industries, or industries at the same time, as shown in Table 1.

**Figure 6:**
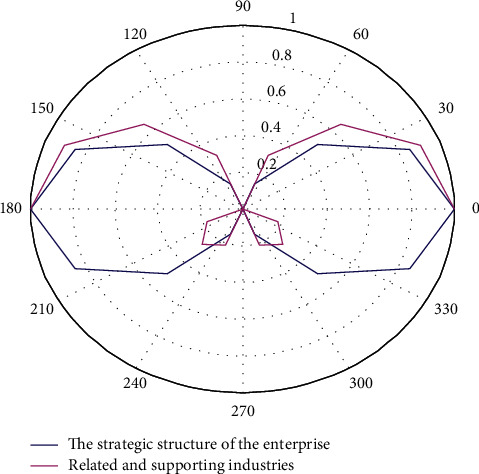
Analysis of China's industrial design industry factors based on the diamond model.

In the era of intelligent interconnection, China's industrial relations and economic structure have undergone drastic changes. Under the new demand and new background of domestic manufacturing development, China's industrial design industry is also facing new plans for industrial structure adjustment and industrial development. In the era of smart interconnection, industrial design will be injected with digital technologies such as smart technology and big data. Industrial design includes the characteristics of innovation and creativity-intensive, knowledge-intensive, and other characteristics. The development of industrial design has a powerful role in promoting innovation in the manufacturing industry and the entire country. Based on the diamond model to analyze China's industrial design industry in the era of intelligent interconnection, it can fully position and analyze the development of the industry, make a long-term plan for the healthy development of China's industrial design industry, and highlight the competitive advantage of China's industrial design industry in the world. The factor analysis of China's industrial design industry based on the diamond model is shown in Figure 6.

**Figure 7:**
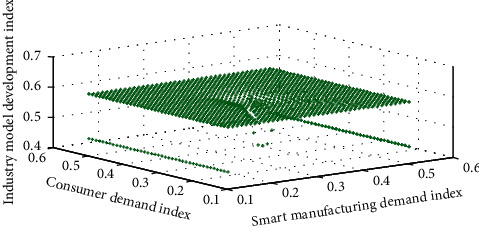
Analysis of the demand conditions of the industrial design industry in the era of intelligent interconnection.

Internet technologies such as the Internet of Things and big data are widely used, making people's lives more convenient, improving people's work efficiency, and changing people's lifestyles. Under the background of this era, the application of industrial design is particularly important. Through industrial design, high-end technology can become more grounded. At the same time, intelligent technology also promotes the development of industrial design. The intervention of the Internet of Things has changed the way of working in traditional industrial design. Therefore, the industrial design industry should make corresponding adjustments and upgrades to meet the new demands of consumers in the era of intelligent interconnection. The demand condition analysis of the industrial design industry in the era of intelligent interconnection is shown in Figure 7.

**Figure 8:**
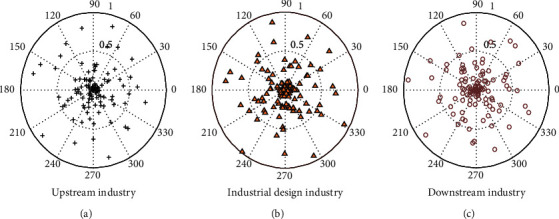
Analysis of related supporting industries in the industrial design industry. (a) Upstream industry. (b) Industrial design industry. (c) Downstream industry.

With the development of social economy, the industrial design industry chain has been gradually improved, and related supporting industries and policy planning have also been further improved. Industrial design has gradually formed a good industrial environment. At present, an industrial design industry composed of upstream industries, industrial design industries, and downstream industries has been formed. Related and supporting industries are shown in Figure 8.

Industrial design consulting companies began to transform to “design house” and other models. This model first appeared in the mobile phone industry. They cooperated with software and hardware suppliers and did not require mass production, and paid more attention to development efficiency. They can develop their own conceptual mobile phones, carry out prototype tests, and modify plans according to customer needs to produce small batches in order to cope with the current market situation with urgent needs at the time and the shortcomings of domestic enterprises with low R&D capabilities. Industrial design has developed rapidly, and enterprise-level industrial design centers have gradually been established. In addition, these industrial design centers will also provide related design services externally (within the industry).

## 6. Conclusion

In this paper, the requirements of IoTs device access system are analyzed in detail from functional and nonfunctional perspectives, and the software system is optimized. Some principles that need to be followed in the design have been designed for the system. Based on the dimensionality reduction processing and ant colony optimization algorithm, a multiterminal selection algorithm ACO-MTS for Internet of Things business is proposed, and it is compared with the random allocation algorithm (RS) and greedy in terms of total business benefits, resource utilization, and business success rate. Compared with GA, the algorithm is improved. However, because the simulation experiment was carried out under several premises and assumptions, it failed to simulate the multiservice batch processing scenario, which still has a gap with the actual IoT multiservice request environment; at the same time, the number of evaluation indicators of the benefit function is relatively less. The next step will be to strengthen simulation experiments for multiservice batch processing scenarios and at the same time design more complex benefit functions to lay the foundation for the optimal allocation of resources in the ubiquitous network peripheral environment of the Internet of Things. The study found that the attribute of industrial design purely as a productive service industry has changed. A large number of designers and design companies are transforming from traditional design services to the design industry under the new situation and new opportunities analyzed and researched in the previous article, that is, from selling ideas at a low price. And the solution to the manufacturer is transformed into the integration of industry chain resources through the information network, creating a better and high-quality life for users, and realizing their own value at the same time.

## Figures and Tables

**Table 1:** Classification of industrial design companies.

Classification based on production objects	Classification based on the main body of production	According to the production content as the standard classification
Companies that provide industrial design services for companies	Industrial design service company	An enterprise centered on physical product design

Companies that provide industrial design services for industries and industries	Industrial design collaboration enterprise	An enterprise focusing on information product design

Art industrial product industry model	Industrial design application enterprise	An enterprise centered on product service system design

## Data Availability

The data used to support the findings of this study are available from the corresponding author upon request.
